# Detection of Unprecedented CYP74 Enzyme in Mammal: Hydroperoxide Lyase CYP74C44 of the Bat *Sturnira hondurensis*

**DOI:** 10.3390/ijms23148009

**Published:** 2022-07-20

**Authors:** Svetlana S. Gorina, Tatiana M. Iljina, Lucia S. Mukhtarova, Yana Y. Toporkova, Alexander N. Grechkin

**Affiliations:** Kazan Institute of Biochemistry and Biophysics, FRC Kazan Scientific Center of RAS, P.O. Box 30, 420111 Kazan, Russia; tatiana.iljina2011@yandex.ru (T.M.I.); lucia74@yandex.ru (L.S.M.); toporkova@kibb.knc.ru (Y.Y.T.)

**Keywords:** cytochrome P450, CYP74, hydroperoxide lyase, mammals, fruit bat, *Sturnira hondurensis*

## Abstract

The genome of the neotropical fruit bat *Sturnira hondurensis* was recently sequenced, revealing an unexpected gene encoding a plant-like protein, CYP74C44, which shares ca. 90% sequence identity with the putative CYP74C of *Populus trichocarpa*. The preparation and properties of the recombinant CYP74C44 are described in the present work. The CYP74C44 enzyme was found to be active against the 13- and 9-hydroperoxides of linoleic and α-linolenic acids (13-HPOD, 13-HPOT, 9-HPOD, and 9-HPOT, respectively), as well as the 15-hydroperoxide of eicosapentaenoic acid (15-HPEPE). All substrates studied were specifically transformed into chain cleavage products that are typical for hydroperoxide lyases (HPLs). The HPL chain cleavage reaction was validated by the identification of NaBH_4_-reduced products (Me/TMS) of 15-HPEPE and 13- and 9-hydroperoxides as (all-*Z*)-14-hydroxy-5,8,11-tetradecatrienoic, (9*Z*)-12-hydroxy-9-dodecenoic, and 9-hydroxynonanoic acids (Me/TMS), respectively. Thus, CYP74C44 possessed the HPL activity that is typical for the CYP74C subfamily proteins.

## 1. Introduction

Cytochromes of the P450 superfamily play numerous roles in the endogenous metabolism of aerobic organisms as well as the control of detoxification of xenobiotics [1,2]. The diversity of P450s is especially high in plants. For example, the rice species *Oryza sativa* (japonica cultivar-group) possesses 356 P450 genes and 99 pseudogenes [3]. The majority of P450s are monooxygenases, oxidizing some hydrophobic substrates and utilizing atmospheric dioxygen as a second substrate [1,2]. All monooxygenases depend on the specific electron transfer chains required for oxygen activation. In contrast to monooxygenases, there are less common non-classical P450s that are not dependent on electron donors and do not need atmospheric oxygen [4,5]. Instead, these P450s control the conversions of peroxides of hydrophobic compounds. For instance, these are the prostacyclin (PGI2) synthase (CYP8A1) and thromboxane (TXA2) synthase (CYP5A1) of mammals that convert the prostaglandin endoperoxides [6]. Plants possess non-classical P450s of a large and diverse CYP74 family, members of which control the conversions of fatty acid hydroperoxides to bioactive oxylipins [7,8]. The CYP74s are the distant congeners of mammalian PGI2 synthase (CYP8A1). The diversity of CYP74 proteins was previously expanded from family to clan after the detection of related genes in proteobacteria, brown algae, and some non-mammalian metazoans, including lancelets [9]. No CYP74 genes have been detected in mammals yet.

The recent sequencing of the bat *Sturnira hondurensis* Goodwin, 1940, genome [10] uncovered the unexpected CYP74 gene, possessing almost 90% identity with the putative CYP74C of the poplar *Populus trichocarpa*. This gene was assigned the name *CYP74C44* (this assignment was generously done by Professor David R. Nelson). This work reports the preparation of the recombinant CYP74C44 of *S. hondurensis* and its identification as a hydroperoxide lyase (HPL).

## 2. Results

### 2.1. Bioinformatics Analyses

**Figure 1:**
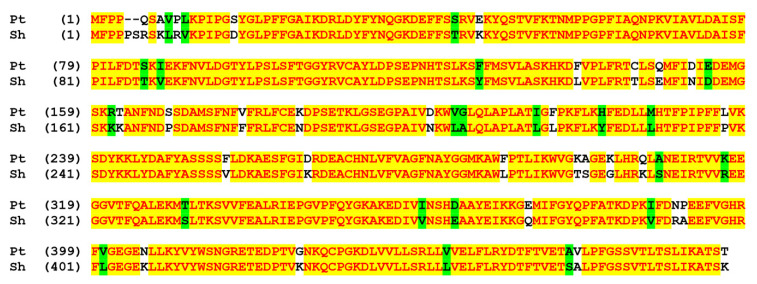
The pairwise alignment of CYP74C44 of *S. hondurensis* (Sh) vs. the putative CYP74C protein of poplar *Populus trichocarpa* (Pt). These two proteins share 88.7% sequence identity. The color scheme reflects the similarity of amino acid sequences.

CYP74C44 of the neotropical fruit bat *S. hondurensis* possesses the greatest kinship with plant proteins of the CYP74C subfamily. The BLAST analyses revealed a high extent of CYP74C44 identity to the putative CYP74C protein XP_002305404.3 of the poplar *Populus trichocarpa* (Figure 1). It also shares about 65.5% identity with the putative CYP74C protein XP_019262969.1 of *Nicotiana attenuata* and CYP74C4 of *Solanum lycopersicum* L. In contrast, CYP74C44 shares only low identity with other P450s of *S. hondurensis*. For example, it has about 16.5% identity with the CYP7B1 (isoform X1, XP_036898685.1) of *S. hondurensis*.

The *CYP74C44* gene is located at the genomic locus LOC118984682. A gene at the nearby LOC118984681 locus encodes a vacuolar-processing enzyme-like sequence possessing a high identity with proteins of different *Populus* species. On the other hand, the adjacent LOC118984680 and LOC118984683 genes encode the elongation factor 1-alpha 1-like protein and adhesion G protein-coupled receptor E2, respectively. The first gene is common for all animal species, while the second one is typical for different bat species.

**Figure 2:**
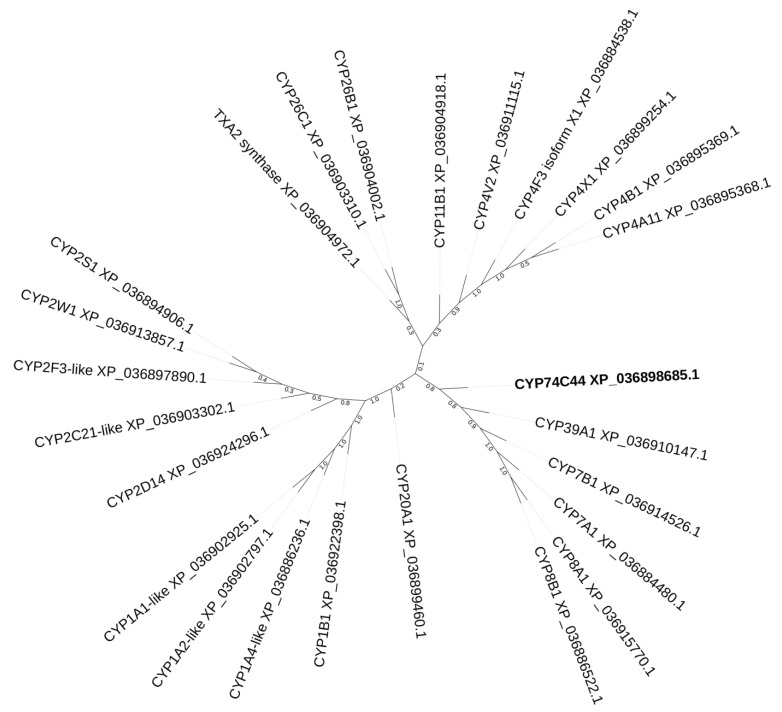
The phylogenetic tree of selected *S. hondurensis* P450s. CYP74C44 is highlighted in bold.

**Figure 3:**
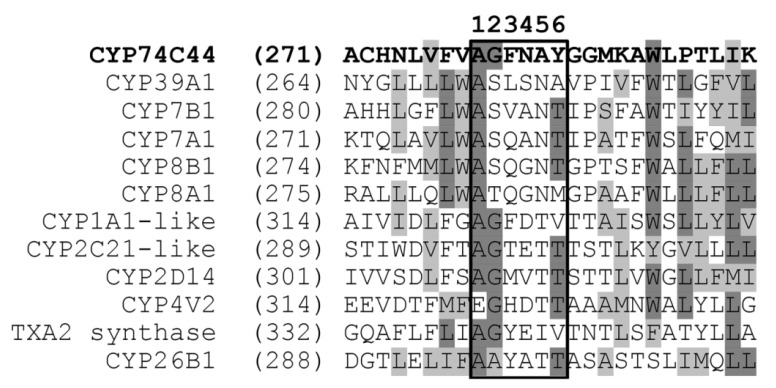
Partial multiple alignment of CYP74C44 vs. the selected P450s of *S. hondurensis* (I-helix region, SRS-4). The I-helix groove motifs are framed.

The construction of a phylogenetic tree of selected *S. hondurensis* P450s (Figure 2) revealed that CYP74C44 is built into a separate branch (Figure 2, left side of the tree), including also the putative prostacyclin PGI2 synthase CYP8A1 and several phylogenetically related proteins, namely CYP8B1, CYP7A1, CYP7B1, and CYP39A1. Another enzyme of prostaglandin endoperoxide metabolism, the putative thromboxane A2 synthase, is built into a separate branch (Figure 2, upper side of the tree) together with the CYP4 and CYP11 proteins. The protein BLAST analyses using CYP74C44 as a query vs. the *S. hondurensis* sequences (the partial multiple alignment of I-helix regions, SRS-4, is presented in Figure 3) showed poor homology. The I-helix groove motif of CYP74C44 possesses some alterations typical for CYP74 enzymes. Firstly, the D/E residue is conserved in monooxygenases such as CYP1A1, CYP2C21, and CYP4V2 (Figure 3, I-helix groove motif, position 4). In contrast, the Asp (N) residue at this position is conserved in all CYP74s, including CYP74C44. The putative PGI2 synthase (CYP8A1) has a Gly (G) residue at this position and an N residue at the next position (5).

To test its biochemical behavior, the recombinant CYP74C44 was incubated with fatty acid hydroperoxides. The results are described below (Section 2.3).

### 2.2. Kinetics and Substrate Specificity of the Recombinant CYP74C44

**Figure 4:**
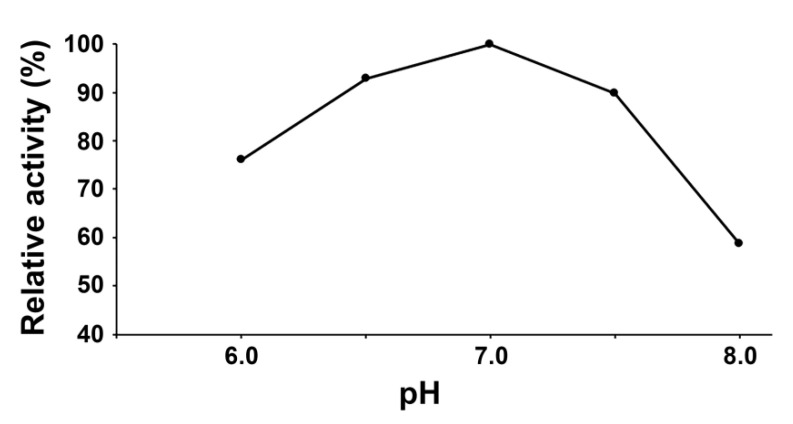
The dependence of the recombinant CYP74C44 catalytic activity on the pH value of the reaction mixture.

The CYP74C44 coding sequence consisted of 1435 nucleotides and encoded a 478 amino acid polypeptide. This sequence was custom synthesized and cloned into the vector pET-23a (Novagen, Madison, WI, USA) to yield the target recombinant protein with a His-tag at the C-terminus. The His-tagged recombinant protein was obtained in BL21-CodonPlus-RIL host strain cells (Stratagene, San Diego, CA, USA) and purified by metal affinity chromatography. The enzymatic activity was controlled using ultraviolet spectroscopy by the decrease in fatty acid hydroperoxide absorbance at 234 nm. The pH optimum of the recombinant CYP74C44 was 7.0 (Figure 4).

The kinetic data revealed that the recombinant CYP74C44 efficiently used 9(*S*)-HPOD, 9(*S*)-HPOT, 13(*S*)-HPOD, 13(*S*)-HPOT, and 15(*S*)-HPEPE as substrates. According to the *K*_m_ values (Table 1), the affinity of CYP74C44 for 15(*S*)-HPEPE was greater than that for α-linolenate and linoleate hydroperoxides. At the same time, the catalytic activity (*k*_cat_) of this enzyme was higher towards linoleate hydroperoxides. However, the CYP74C44 enzyme exhibited the highest catalytic efficiency for C20 hydroperoxides (3–6 times higher than C18 hydroperoxides).

### 2.3. Substrate and Product Specificities of the Recombinant CYP74C44

**Figure 5:**
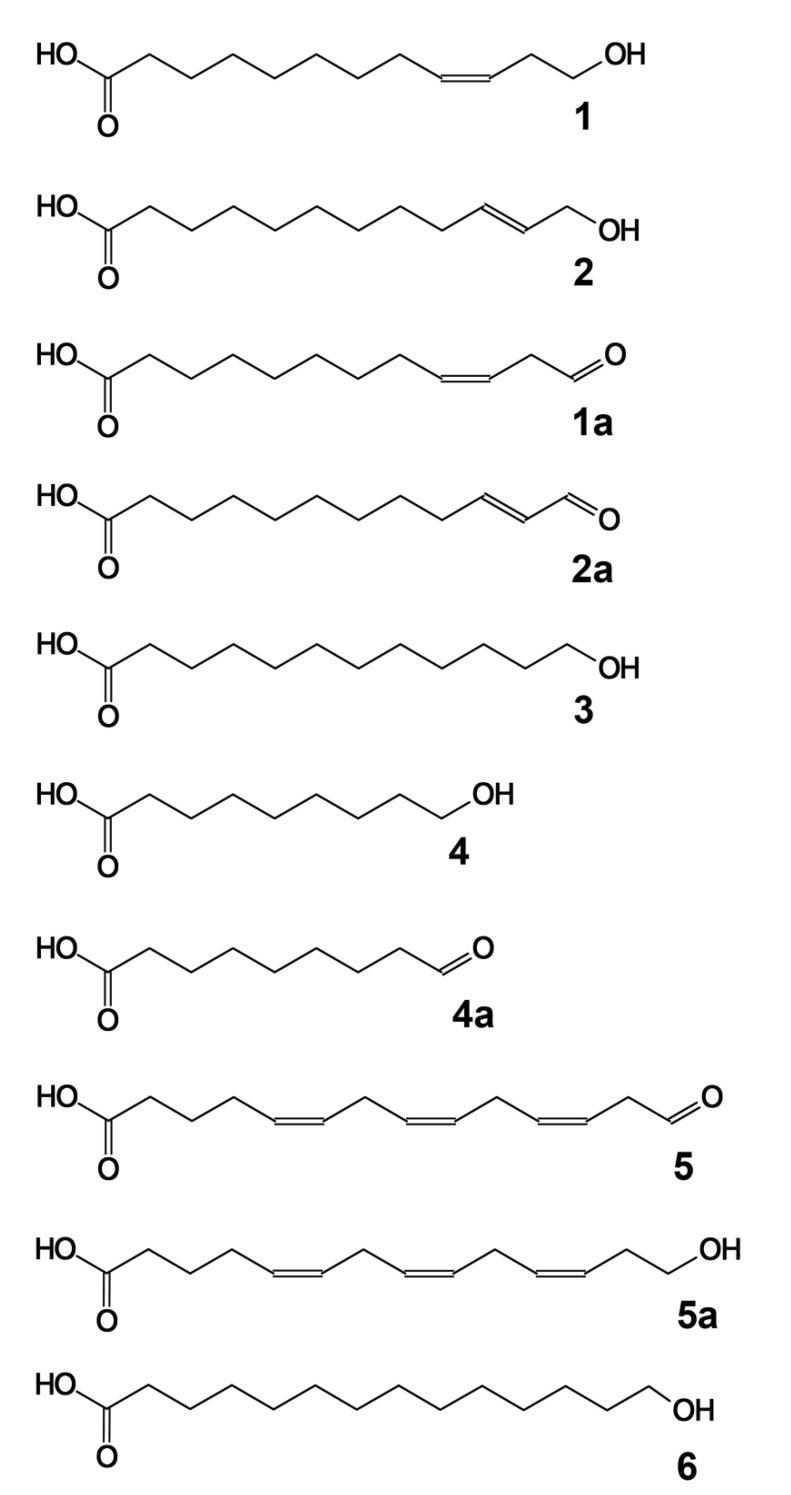
The structural formulae of major detected products and their derivatives: **1**, (9*Z*)-12-hydroxy-9-dodecenoic acid; **1a**, (9*Z*)-12-oxo-9-dodecenoic acid; **2**, (10*E*)-12-hydroxy-10-dodecenoic acid; **3**, 12-hydroxydodecanoic acid; **4**, 9-hydroxynonanoic acid; **4a**, 9-oxononanoic acid; **5**, 14-hydroxy-5,8,11-tetradecatrienoic acid; **5a**, (all-*Z*)-14-oxo-5,8,11-tetradecatrienoic acid; **6**, 14-hydroxytetradecanoic acid.

The recombinant CYP74C44 was incubated with the 13- and 9-hydroperoxides of linoleic and α-linolenic acids (13-HPOD, 13-HPOT, 9-HPOD, and 9-HPOT, respectively), as well as the 15-hydroperoxide of eicosapentaenoic acid (15-HPEPE). The products (Me/TMS, with or without preliminary NaBH_4_ reduction) were subjected to GC–MS analyses. The GC–MS chromatograms of the NaBH_4_-reduced products (Me/TMS) are presented in Figure 5. The structural formulae of the products formed by the recombinant enzyme are also presented in Figure 5.

**Figure 6:**
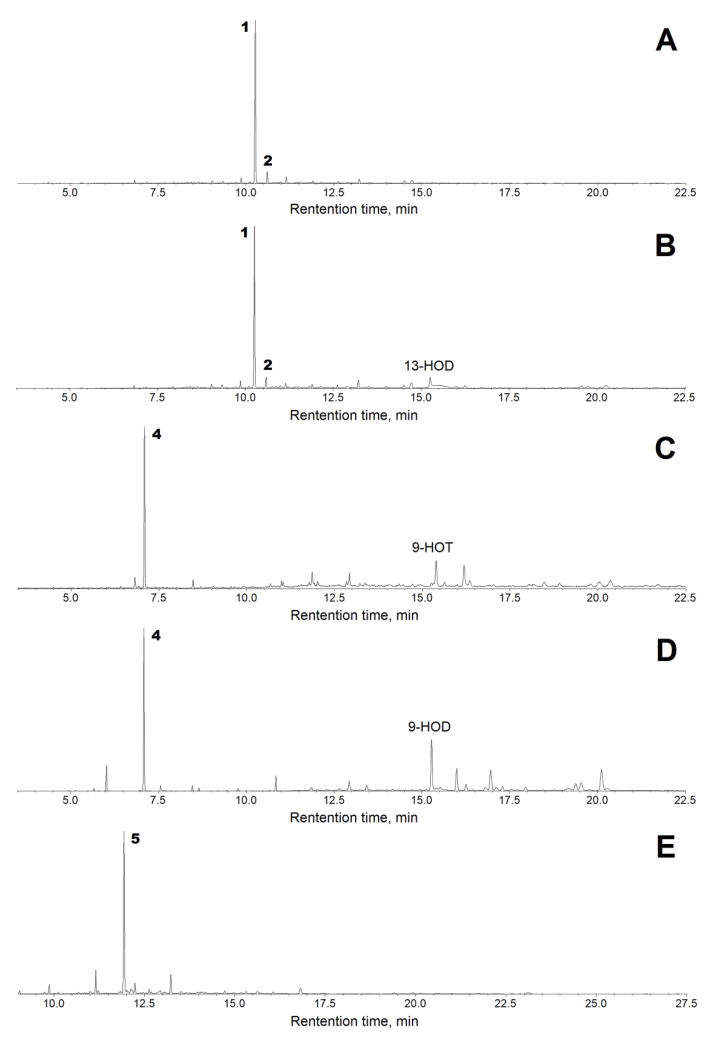
The total ion current GC–MS chromatograms of the products (Me/TMS) of incubation of 13-HPOT (**A**), 13-HPOD (**B**), 9-HPOT (**C**), 9-HPOD (**D**), and 15-HPEPE (**E**) with recombinant CYP74C44 of *S. hondurensis.* Conditions of incubation, extraction, derivatization, and analysis are described in Section 4. Abbreviations: 13-HOD, (9Z,11E,13S)-13-hydroxy-9,11-octadecadienoic acid; 9-HOT, (9S,10E,12Z,15Z)-9-hydroxy-10,12,15-octadecatrienoic acid; 9-HOD, (9S,10E,12Z)-9-hydroxy-10,12-octadecadienoic acid.

Both 13-HPOT and 13-HPOD incubations yielded a single predominant product, **1**, with a retention time of 10.3 min (Figure 6A,B). The mass spectrum of this NaBH_4_-reduced product (Me/TMS) possessed the following diagnostic and prominent fragments: [M–Me]^+^ at *m*/*z* 285 (1%), [M–CH_2_OTMS + TMS]^+^ at *m*/*z* 270 (5%), [285–MeOH]^+^ at *m*/*z* 253 (6%), [M–TMSOH]^+^ at *m*/*z* 210 (5%), [210–MeOH]^+^ at *m*/*z* 178 (13%), *m*/*z* 159 (12%), *m*/*z* 123 (31%), [CH_2_=O^+^–SiMe_3_] at *m*/*z* 103 (100%), and [SiMe_3_]^+^ at *m*/*z* 73 (86%). The spectrum of compound **1** matched that for (9*Z*)-12-hydroxy-9-dodecenoic acid (Me/TMS) [11]. The next eluting smaller peak for **2** with a retention time of 10.3 min possessed similar spectral patterns: M^+^ at *m*/*z* 300 (0.6%), [M–Me]^+^ at *m*/*z* 285 (2%), [285–MeOH]^+^ at *m*/*z* 253 (13%), *m*/*z* 185 (4%), *m*/*z* 159 (8%), *m*/*z* 143 (12%), *m*/*z* 129 (64%), *m*/*z* 75 (47%), and [SiMe_3_]^+^ at *m*/*z* 73 (100%). The spectrum of product **2** corresponded to that for (10*E*)-12-hydroxy-10-dodecenoic acid (Me/TMS) [11]. Catalytic hydrogenation of both compounds **1** and **2** (Me esters) followed by trimethylsilylation resulted in the product **3** (Me/TMS), whose mass spectrum possessed M^+^ at *m*/*z* 302 (0.6%), [M–Me]^+^ at *m*/*z* 287 (15%), [287–MeOH]^+^ at *m*/*z* 255 (100%), *m*/*z* 204 (28%), *m*/*z* 191 (12%), *m*/*z* 107 (20%), [CH_2_=O^+^–SiMe_3_] at *m*/*z* 103 (22%), *m*/*z* 75 (48%), and [SiMe_3_]^+^ at *m*/*z* 73 (62%). This spectrum matched that for 12-hydroxydodecanoic acid (Me/TMS) [11]. Overall, the described data allowed us to ascribe the structures of (9*Z*)-12-hydroxy-9-dodecenoic acid (Me/TMS) and (10*E*)-12-hydroxy-10-dodecenoic acid (Me/TMS) to compounds **1** and **2**, respectively. Thus, the obtained data indicated that the primary CYP74C44 product was an aldoacid (9*Z*)-12-oxo-9-dodecenoic acid (**1a**), which was detectable during the GC–MS analyses of unreduced products (data not illustrated). However, the (9*Z*)-12-oxo-9-dodecenoic acid (Me) is hardly detectable due to low ionization. Therefore, the 13-HPL products were analyzed after NaBH_4_ reduction.

The GC–MS analyses of 9-HPOT and 9-HPOD products (Me/TMS) revealed compound **4a**, which exhibited the following mass spectral patterns: M^+^ at *m*/*z* 186 (0.03%), [M–C=O]^+^ at *m*/*z* 158 (8%), [M–MeOH]^+^ at *m*/*z* 155 (16%), [M–CH_2_CH=O]^+^ at *m*/*z* 143 (16%), *m*/*z* 111 (56%), *m*/*z* 87 (74%), *m*/*z* 83 (60%), *m*/*z* 74 (100%), and *m*/*z* 69 (31%). The spectrum matched that for 9-oxononanoic acid (Me) [11]. Analyses of NaBH_4_-reduced 9-HPOT and 9-HPOD products (Me/TMS) showed a predominance of the single product **4**, possessing the following mass fragmentation patterns: [M–H]^+^ at *m*/*z* 259 (0.1%), [M–Me]^+^ at *m*/*z* 245 (20%), [245–MeOH]^+^ at *m*/*z* 213 (100%), *m*/*z* 195 (4%), *m*/*z* 171 (6%), [CH_2_=O^+^–SiMe_3_] at *m*/*z* 103 (29%), *m*/*z* 89 (35%), *m*/*z* 75 (47%), and [SiMe_3_]^+^ at *m*/*z* 73 (56%). The spectrum matched that for 9-hydroxynonanoic acid (Me/TMS) [11]. Thus, the obtained data supported the identification of the major detectable primary product **4a** of 9-HPOT and 9-HPOD conversions as 9-oxononanoic acid, the HPL chain cleavage product. The conversion of 9-HPOD was less efficient compared with that of 9-HPOT. Part of 9-HPOD remained unused (Figure 6D compared to Figure 6C). The 9-HPOD product profiling (Figure 6D) revealed some side formation of the epoxy alcohol 9,10-epoxy-11-hydroxy-12-octadecenoic acid (Me/TMS) besides the 9-oxononanoic acid (Me).

The GC–MS profile of NaBH_4_-reduced products (Me/TMS) of enzyme incubation with 15-HPEPE showed a single predominant product **5**, whose mass spectrum possessed M^+^ at *m*/*z* 324 (0.1%), [M–Me]^+^ at *m*/*z* 309 (0.5%), [309–MeOH]^+^ at *m*/*z* 277 (0.2%), [M–TMSOH]^+^ at *m*/*z* 234 (3%), *m*/*z* 185 (2%), *m*/*z* 160 (7%), *m*/*z* 133 (13%), *m*/*z* 119 (46%), [CH_2_=O^+^–SiMe_3_] at *m*/*z* 103 (60%), and [SiMe_3_]^+^ at *m*/*z* 73 (100%). A prominent fragment at *m*/*z* 103 indicated the presence of a primary alcohol (TMS) function. These fragmentation patterns supported the structure of 14-hydroxy-5,8,11-tetradecatrienoic acid (Me/TMS) for compound **5**. Furthermore, the catalytic hydrogenation over PtO_2_ turned compound **5** into the saturated analogue **6** (Me/TMS), which possessed the following mass fragmentation pattern: M^+^ at *m*/*z* 330 (0.1%), [M–Me]^+^ at *m*/*z* 315 (15%), [315–MeOH]^+^ at *m*/*z* 283 (100%), *m*/*z* 185 (2%), *m*/*z* 159 (6%), *m*/*z* 146 (7%), [CH_2_=O^+^–SiMe_3_] at *m*/*z* 103 (17%), *m*/*z* 75 (36%), and [SiMe_3_]^+^ at *m*/*z* 73 (29%). The hydrogenation increased the molecular mass from 324 (compound **5**) to 330 (hydrogenation product), thus indicating the presence of three double bonds in compound **5**. The last spectrum allowed for ascribing a structure of 14-hydroxytetradecanoic acid (Me/TMS) to the hydrogenation product and a structure of 14-hydroxy-5,8,11-tetradecatrienoic acid (Me/TMS) to compound **5**. Overall, the described data indicated that the (all-*Z*)-14-oxo-5,8,11-tetradecatrienoic acid (**5a**) was formed as the product of 15-HPEPE conversion. Based on the results obtained, the name ShHPL (*S. hondurensis* hydroperoxide lyase) has been assigned to CYP74C44 and the name *ShHPL* to the corresponding gene.

## 3. Discussion

CYP74C44 appears to be a typical plant CYP74 protein, with up to 90% identity to some CYP74C subfamily members. The CYP74C subfamily includes both HPL [12,13,14,15,16,17,18,19,20] and AOS [21,22,23,24] members. Thus, the identification of recombinant CYP74C44 as an unspecific HPL is not surprising. The majority of the CYP74C proteins studied thus far belong to Solanaceae (Asterids). No CYP74 enzymes have been detected in mammals before. Only non-mammalian metazoans such as lancelets [9,25], sea anemones [26,27], and stony corals [9] have been shown to possess the CYP74 clan enzymes.

For a long time, hydroperoxide lyase (HPL) has been proposed to control the chain cleavage of fatty acid hydroperoxides [12]. However, more recent studies have revealed that the enzyme is in fact an isomerase that converts the fatty acid hydroperoxides into a short-lived hemiacetal, spontaneously decomposing into two aldehyde fragments [17,28,29]. In view of these findings, the synonymous name “hemiacetal synthase”, corresponding to the true catalytic function, was proposed [29]. Aldehydes and aldoacids, the products of hemiacetal decomposition, play a defensive and regulatory role in plants [12,13]. HPL products such as (2*E*)-hexenal (“leaf aldehyde”) may cause gene damage by their addition to deoxyguanosine residues of DNA [30] and exhibit general genotoxicity, cytotoxicity, and antimicrobial and fungicidal activity [12,13]. Interestingly, recent research [31,32] demonstrated the fungicidal effects of exogenous (2*E*)-hexenal (“leaf aldehyde”) against the psychrophilic fungus *Pseudogymnoascus destructans*, which causes the white-nose syndrome disease in bats. The results of the present work showed the presence of an endogenous HPL, a key enzyme of aldehyde biosynthesis, in *S. hondurensis.* The antifungal resistance might justify the physiological requirements of the HPL genes in the bats. However, the mechanism of the proposed quite uncommon gene transfer from plant to mammal remains to be revealed. Further genomic sequencing of the bats is needed to shed more light on this phenomenon.

**Figure 7:**
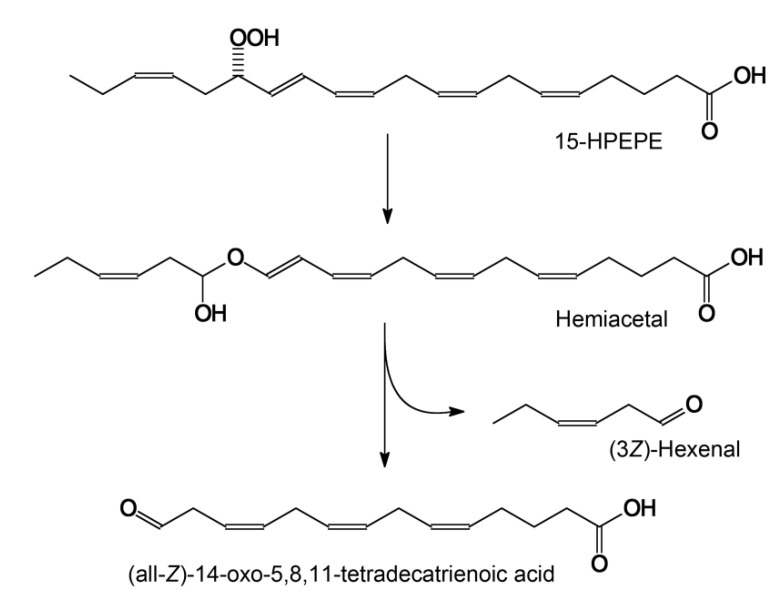
The mechanisms of 15(*S*)-HPEPE conversions by ShHPL to major product—(all-*Z*)-14-oxo-5,8,11-tetradecatrienoic acid.

The SciFinder search revealed no mentions of (all-*Z*)-14-oxo-5,8,11-tetradecatrienoic acid as an HPL product in the literature. This compound has only been described as a product of chemical [33] or combined enzymatic–chemical [34] conversions of arachidonic acid. The loss of information on C_14_ aldoacid production by HPLs is not surprising since 15-HPEPE and 15-HPETE are not the physiological substrates for plant HPLs. The occurrence of HPL in the bat creates prerequisites for biosynthetic pathways from eicosapentaenoic or arachidonic acids to (all-*Z*)-14-oxo-5,8,11-tetradecatrienoic acid (see the mechanistic scheme in Figure 7). Moreover, 15-HPEPE is the preferred substrate for CYP74C44.

The detection of a typical *CYP74C* gene in the bat genome appears surprising. The *CYP74C44* gene is highly homologous to the CYP74s of poplar and other plant species but has only weak homology to other P450s of *S. hondurensis*. These facts raise the question of the origin of the *CYP74C44* gene in the *S. hondurensis* genome. Since *S. hondurensis* is a fruit-feeding bat, one can propose that the *CYP74C44* gene could emerge from fruits. However, the BLAST search revealed another CYP74 family gene (CYP74B) in the genome of the distinct bat species *Rhinolophus ferrumequinum* (information generously communicated by Professor David R. Nelson). This species is insect-feeding. Thus, the food could not serve as a direct source of a “plant gene” in this species. Moreover, the genes located at adjacent loci encode proteins that are typical for animals but not for plants. Further studies of bat genomes are needed to resolve the intriguing question of the origin of these genes.

Concluding remarks: (1) The neotropical fruit bat *S. hondurensis* has the plant-like protein CYP74C44, possessing ca. 90% sequence identity with the putative CYP74C of *Populus trichocarpa*. (2) The recombinant CYP74C44 was active towards the 13- and 9-hydroperoxides of linoleic and α-linolenic acids as well as the 15-hydroperoxide of eicosapentaenoic acid; the last one was the preferred substrate. (3) The recombinant CYP74C44 possessed hydroperoxide lyase (HPL) activity towards all tested fatty acid hydroperoxides and converted them into the chain cleavage products, aldehydes and aldoacids. (4) Presumably, CYP74C44 and its products may play a defensive role against fungi infecting the bats.

## 4. Materials and Methods

### 4.1. Materials

Linoleic, α-linolenic, and eicosapentaenoic acids, as well as the soybean lipoxygenase type V, were purchased from Sigma. NaBH_4_ and silylating reagents were purchased from Fluka (Buchs, Switzerland). (9*S*,10*E*,12*Z*)-9-Hydroperoxy-10,12-octadecadienoic (9-HPOD) and (9*S*,10*E*,12*Z*,15*Z*)-9-hydroperoxy-10,12,15-octadecatrienoic (9-HPOT) acids were prepared by incubation of linoleic and α-linolenic acids, respectively, with recombinant maize 9-lipoxygenase (GenBank: AAG61118.1) [35] at 0 °C and Na phosphate buffer (100 mM, pH 6.0) under continuous oxygen bubbling. For the preparation of (9*Z*,11*E*,13*S*)-13-hydroperoxy-9,11-octadecadienoic (13-HPOD), (9*Z*,11*E*,13*S*,15*Z*)-13-hydroperoxy-9,11,15-octadecatrienoic (13-HPOT), and (5*Z*,8*Z*,11*Z*,13*E*,15*S*,17*Z*)-13-hydroperoxy-5,8,11,13,17-eicosapentaenoic (15-HPEPE) acids, linoleic, α-linolenic, and eicosapentaenoic acids, respectively, were incubated with the soybean lipoxygenase type V at 23 °C and Tris-HCl buffer (50 mM, pH 9.0) under continuous oxygen bubbling. The extracted hydroperoxides (as free carboxylic acids) were purified by normal phase HPLC (NP-HPLC) on a Macherey-Nagel Nucleodur 100-3 silica column (250 × 4.6 mm, 3 μm) under isocratic elution with the solvent mixture hexane/isopropanol/acetic acid (98.1:1.8:0.1, by volume) at a flow rate of 0.4 mL/min. Hydroperoxides were chromatographically pure and at least 98% optically pure, as judged by chiral phase HPLC [36].

### 4.2. Bioinformatic Methods

The search for CYP74-related genes was carried out in the NCBI database. Primer construction was performed using the Vector NTI Advance 11.5 program (Invitrogen, Madison, WI, USA). The BLAST analyses of the CYP74s were performed using the protein NCBI BLAST tool. The multiple alignments of selected CYP74 amino acid sequences were made with Clustal Omega and MEGA7 software [37]. The phylogenetic analysis was carried out using the maximum likelihood method based on the Poisson correction model [38], and the bootstrap consensus tree was inferred from 1000 replicates. The iTOL tool (https://itol.embl.de/ (accessed on 1 February 2022)) was used to visualize the phylogenetic model output.

### 4.3. Expression and Purification of Recombinant Enzyme

The target sequence encoding the CYP74C44 sequence was synthesized by the Lumiprobe Company (Saint Petersburg, Russia). The plasmid containing the target sequence was subsequently cut with NdeI and XhoI and subcloned into the same sites of the expression vector pET-23a (Novagen, Madison, WI, USA) to yield the target recombinant protein with a His-tag at the C-terminus. The resulting construction was transformed into the *Escherichia coli* host strain BL21-CodonPlus-RIL (Stratagene, San Diego, CA, USA). Using the pET-23a vector allowed the preparation of the target recombinant protein with a His-tag at the C-terminus. The resulting construction was sequenced to verify the presence of the CYP74C44 sequence. The recombinant gene was expressed in host cells as described before [39]. Purification of the His-tagged recombinant protein was performed using a Bio-Scale Mini Profinity IMAC (immobilized metal affinity chromatography) cartridge in the BioLogic LP chromatographic system (Bio-Rad, Hercules, CA, USA) (Supplementary Figure S1). The recombinant enzyme was eluted from the cartridges using 50 mM histidine. The homogeneity of the purified protein was confirmed by SDS-PAGE. The protein concentration was estimated as described before [40].

### 4.4. Kinetic Studies

The enzymatic activity of the purified recombinant CYP74C44 was determined by monitoring the 234 nm signal decrease with a PB 2201 B UV-VIS spectrophotometer (SOLAR, Minsk, Belarus) with substrate concentrations ranging from 5 to 150 μM. The measurements were carried out in 0.6 mL of Na phosphate buffer (pH 7.0) at 25 °C. A molar extinction coefficient of 25,000 M^−1^ cm^−1^ at 234 nm for fatty acid hydroperoxides was used. Kinetic parameters were calculated by fitting the datasets to a one-site saturation model for simple ligand binding using the SigmaPlot 11 software (Systat Software Inc., Palo Alto, CA, USA). Five independent experiments were performed for each specified variant.

### 4.5. Incubations of Recombinant Enzyme with Substrates

The recombinant enzyme (10 μg) was incubated with 100 μg of 9-HPOD, 9-HPOT, 13-HPOD, 13-HPOT, or 15-HPEPE for 15 min at 23 °C in 10 mL of Na phosphate buffer (100 mM, pH 7.0). The reaction mixture was acidified to pH 6.0, and the products were extracted with a hexane/ethyl acetate (1:1, by volume) mixture. The products were methylated with ethereal diazomethane and trimethylsilylated with a pyridine/hexamethyldisilazane/trimethylchlorosilane (2:1:2, by volume) mixture at 23 °C for 15 min. The silylation reagents were evaporated in vacuo. The dry residue was dissolved in 100 μL hexane and subjected to GC–MS analyses. When specified, the products were reduced with NaBH_4_, then methylated and trimethylsilylated. In separate cases, the products (Me esters) were subjected to hydrogenation over PtO_2_, followed by trimethylsilylation. The product derivatives were analyzed as Me esters/TMS derivatives (Me/TMS) by GC–MS.

### 4.6. Methods of Spectral Analyses

The products were analyzed as Me/TMS derivatives by GC–MS as described before [17]. The GC–MS analyses were performed using a Shimadzu QP2020A mass spectrometer connected to a Shimadzu GC-2010 Plus gas chromatograph equipped with a Macherey-Nagel Optima-5-MS (5% phenyl, 95% methylpolysiloxane) fused capillary column (length, 30 m; ID, 0.25 mm; film thickness, 0.25 μm). Helium at a linear velocity of 30 cm/s was used as the carrier gas. Injections were made in the split mode using an initial column temperature of 120 °C and an injector temperature of 230 °C. Then, the column temperature was raised at 10 °C/min until 240 °C. Electron impact ionization (70 eV) was used.

## Figures and Tables

**Table 1:** Kinetic parameters and substrate specificities of the recombinant CYP74C44.

Substrate	*k*_cat_ (s^−1^)	*K*_m_ (μM)	*k*_cat_/*K*_m_ (μM^−1^∙s^−1^)	Catalytic Efficiency, % 15(*S*)-HPEPE
15-HPEPE	275 ± 16	8.17 ± 1.6	33.7	100
9-HPOT	254.2 ± 26	20.9 ± 5.6	12.2	36.2
9-HPOD	551.3 ± 19	46.6 ± 7.2	11.8	35
13-HPOT	189 ± 18	28.4 ± 6.7	6.7	19.9
13-HPOD	291.8 ± 28	57.9 ± 13.3	5.1	15.1

## Data Availability

Not applicable.

## Supplementary Materials

The following supporting information can be downloaded at: https://www.mdpi.com/article/10.3390/ijms23148009/s1.
