# Supplementary material for: Detection of Unprecedented CYP74 Enzyme in Mammal: Hydroperoxide Lyase CYP74C44 of the Bat Sturnira hondurensis
Source: Int J Mol Sci. 2022 Jul 20;23(14):8009. doi: 10.3390/ijms23148009 (PMC9320521; doi:10.3390/ijms23148009)
Supplement: Supplementary file 1 [file ijms-23-08009-s001.zip › ijms-1814988-supplementary.pdf]

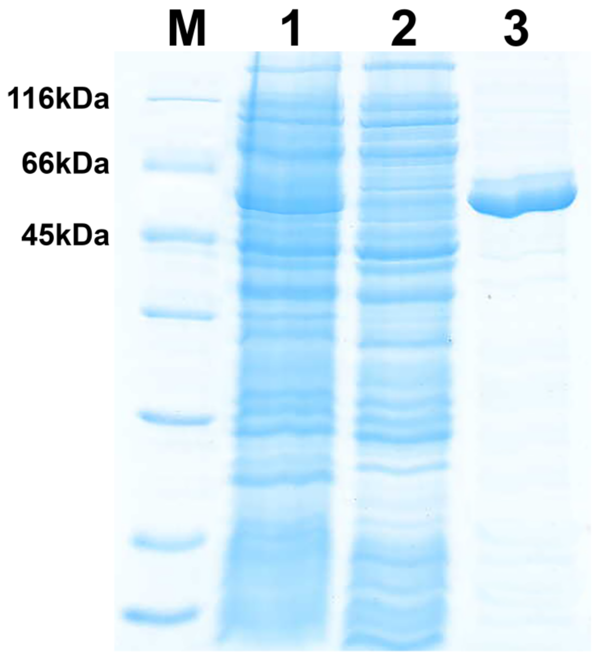

Supplementary Figure S1. SDS-PAGE analysis of expressing and purifying CYP74C44 protein: M, SDS-PAGE standards Low Range Protein Ladder (Bio-Rad, USA); 1, whole cell sample of strain BL21-CodonPlus-RIL 16 hours after IPTG addition; 2, whole cell sample of strain BL21-CodonPlus-RIL before induction with IPTG; 3, purified and concentrated recombinant protein.
